# ^1^Selenium supply alters the subcellular distribution and chemical forms of cadmium and the expression of transporter genes involved in cadmium uptake and translocation in winter wheat (*Triticum aestivum*)

**DOI:** 10.1186/s12870-020-02763-z

**Published:** 2020-12-07

**Authors:** Jiaojiao Zhu, Peng Zhao, Zhaojun Nie, Huazhong Shi, Chang Li, Yi Wang, Shiyu Qin, Xiaoming Qin, Hongen Liu

**Affiliations:** 1 Resources and Environment College, Henan Agricultural University, No. 63, Nongye Road, Jinshui District, Zhengzhou, 450002 Henan Province China; 2 Department of Chemistry and Biochemistry, Texas Tech University, Lubbock, TX 79409 USA

**Keywords:** Selenium, Cadmium, Subcellular distribution, Chemical forms, Gene expression, Wheat

## Abstract

**Background:**

Cadmium (Cd) accumulation in crops affects the yield and quality of crops and harms human health. The application of selenium (Se) can reduce the absorption and transport of Cd in winter wheat.

**Results:**

The results showed that increasing Se supply significantly decreased Cd concentration and accumulation in the shoot and root of winter wheat and the root-to-shoot translocation of Cd. Se application increased the root length, surface area and root volume but decreased the average root diameter. Increasing Se supply significantly decreased Cd concentration in the cell wall, soluble fraction and cell organelles in root and shoot. An increase in Se supply inhibited Cd distribution in the organelles of shoot and root but enhanced Cd distribution in the soluble fraction of shoot and the cell wall of root. The Se supply also decreased the proportion of active Cd (ethanol-extractable (FE) Cd and deionized water-extractable (FW) Cd) in root. In addition, the expression of *TaNramp5*-a, *TaNramp5*-b, *TaHMA3*-a, *TaHMA3*-b and *TaHMA2* significantly increased with increasing Cd concentration in root, and the expression of *TaNramp5-a*, *TaNramp5-b* and *TaHMA2* in root was downregulated by increasing Se supply, regardless of Se supply or Cd stress. The expression of *TaHMA3*-b in root was significantly downregulated by 10 μM Se at both the 5 μM and 25 μM Cd level but upregulated by 5 μM Se at the 25 μM Cd level. The expression of *TaNramp5*-a, *TaNramp5*-b, *TaHMA3*-a, *TaHMA3*-b and *TaHMA2* in shoot was downregulated by increasing Se supply at 5 μM Cd level, and 5 μM Se upregulated the expression of those genes in shoot at 25 μM Cd level.

**Conclusions:**

The results confirm that Se application limits Cd accumulation in wheat by regulating the subcellular distribution and chemical forms of Cd in winter wheat tissues, as well as the expression of *TaNramp5-a*, *TaNramp5*-b and *TaHMA2* in root.

**Supplementary Information:**

The online version contains supplementary material available at 10.1186/s12870-020-02763-z.

## Background

Cadmium (Cd) is one of the most dangerous heavy metals due to its detrimental effects on agricultural soil and potential harm to human health [1]. It is generally believed that plants are the major source of Cd uptake by humans. Thus, Cd can harm human health through the enrichment effect of the food chain. Wheat is not only one of the principal foods in northern China but also the most important grain crop in the world [2]. Cd-polluted wheat accumulation in humans may cause many diseases, such as anemia, osteoporosis, kidney damage and hypertension [3]. Therefore, it has become an urgent public health problem to reduce the accumulation of Cd in wheat and maintain food safety [4].

Although Cd has no essential biological function in plants, the accumulation of Cd in plants can produce obvious toxic effects, including destroying chlorophyll, inhibiting photosynthesis and crop growth and development, and reducing yield and quality [5]. The intracellular and extracellular mechanisms for detoxification in plants have gradually developed in the process of adapting to heavy metal stress. Binding in the cell wall and transfer to vacuoles may be associated with metal tolerance [6]. The toxicity and migration ability of heavy metals are closely related to their chemical forms. This suggests that Cd chemical forms may affect the movement of Cd in plants and may be one of the major mechanisms of heavy metal detoxification [7]. The total amount of Cd entering plants is determined by the absorption capacity of Cd in root. Cd in soil is absorbed by plant root and transported to other parts of the plant through transporters for essential elements, such as manganese (Mn), zinc (Zn) and iron (Fe) [8]. At least seven families of transporters participate in Cd transport in plants, including natural resistance-associated macrophage proteins (NRAMP), heavy metal ATPases (HMA), ATP-binding cassette transporters (ABC), Zrt/Irt-like proteins (ZIP), H^+^/cation exchanger (CAX), LCT transporter and the cation efflux family (CE) [9]. Se is an essential trace element for humans, animals and plants [10]. Se can promote the growth and development of plants by improving antioxidant function and regulating photosynthesis. In addition, Se plays a vital role in plant resistance to adverse stress and the alleviation of the toxicity of heavy metals [11]. Se is also a beneficial element for humans and can maintain human health by improving immunity, resisting aging and reducing cancer risk [12]. In recent years, many research results have shown that Se and Cd in plants are antagonistic. Sun et al. [13] found that Se could reduce Cd concentrations in maize and promote maize growth under Cd stress. Wan et al. [14] also reported that the translocation of Cd from root to shoot decreased effectively with increasing Se supply in rice seedlings. In addition, Ahmad et al. [15] found that Se reduces Cd toxicity by regulating the antioxidative system in *Brassica juncea*. Shanker et al. [16] revealed that Se and Cd can be combined to form a complex, thus reducing the toxicity of Cd. These studies suggest that applying Se fertilizer is an effective way to reduce Cd accumulation in plants.

The aims of the present study were to i) re-examine the effects of different Se supply rates on Cd uptake and translocation; ii) investigate the subcellular distribution and chemical forms of Cd in response to different Se supply rates; and iii) investigate the expression of Cd transporter genes regulated by different Se supply rates under two levels of Cd stress using a hydroponic trial. Our results will contribute to a better understanding of the mechanisms by which Se inhibits Cd uptake and translocation in winter wheat.

## Methods

### Plant materials and experimental design

Winter wheat (*Triticum aestivum* cv Zhengmai379, obtained from Henan Agricultural High Tech Group Co., Ltd.) seeds were sterilized for 15 min with 10% NaClO, rinsed with deionized water, and then cultured at 25 °C for 5 days. Then, 20 seedlings of the same size were transferred to plastic pots containing 4 L of nutrient solution. The composition of the nutrient solution was 6.0 mM KNO_3_, 4.0 mM Ca (NO_3_)_2_·4H_2_O, 2.0 mM MgSO_4_·7H_2_O, 1.0 mM NaH_2_PO_4_·2H_2_O, 100 μM EDTA-Fe, 46 μM H_3_BO_3_, 9 μM MnCl_2_·4H_2_O, 0.8 μM ZnSO_4_·7H_2_O, 0.3 μM CuSO_4_·5H_2_O, and 0.09 μM Na_2_MoO_4_·2H_2_O. Cd was added to the solution as CdCl_2_ at two levels: 5 and 25 μM, and Se was added as Na_2_SeO_3_ at three levels: 0, 5, and 10 μM after seedlings were transferred for 1 week. Six treatments were included: Cd_5_Se_0_, Cd_5_Se_5_, Cd_5_Se_10_, Cd_25_Se_0_, Cd_25_Se_5_ and Cd_25_Se_10_. Each treatment was replicated three times. The quarter- and half-strength nutrient solutions were provided in the first and second weeks, respectively, followed by full-strength nutrient solutions. The greenhouse conditions were as follows: relative humidity 70%, 14 h light/10 h dark at 25/18 °C, and light intensity of 400 μmol m^− 2^ s^− 1^.

The seedlings were harvested after 21 days, and the shoot and root were separated. Ten seedlings were taken from each pot, and the root were soaked in 0.5 mM CaCl_2_ and 2 mM MES solution for 30 min, washed with deionized water three times, and then dried in an electric oven at 60 °C until the weight was constant to analyze dry matter weight and Cd concentration in plant issues. The others were frozen in liquid nitrogen immediately and then stored at − 80 °C for further subcellular fraction, chemical form and gene expression analysis.

### Determination of Cd concentration

Cd concentrations in plant tissues were determined by the method described by Liu et al. [17]. Dry samples were powdered and digested in a mixture of HNO_3_:HClO_4_ (4:1, v/v). The Cd concentration in solution was determined using a flame atomic absorption spectrophotometer (ZEEnit 700, Analytik Jena AG, Germany).

### Determination of root morphology

After 14 days of seedling growth, one seedling from each pot was taken for root morphological analysis. The root length, root surface area, root volume, and average root diameter of the wheat samples were measured by using the root imaging analysis software WinRHI-ZO Version 2009 PRO (Regent Instruments, Quebec City, Canada).

### Determination of subcellular fractions

Using methods described by Zhao et al. [18], frozen samples were homogenized in precooled extraction buffer containing 50 mM Tris-HCl (pH 7.5), 1.0 mM dithiothreitol (C_4_H_10_O_2_S_2_) and 250 mM sucrose at a ratio of 1:20 (w/v). The mixtures were centrifuged at 924×*g* for 15 min, and the cell wall fraction was obtained in the pellet. After centrifuging the supernatant at 20,000×*g* for 45 min, the supernatant solution and precipitate were called the soluble fraction and cell organelle fraction, respectively. All steps were carried out at 4 °C. A mixture of HNO_3_:HClO_4_ (4:1, v/v) was used for wet digestion of the different fractions, and the Cd concentration in the digestion solution was determined using a flame atomic absorption spectrophotometer (ZEEnit 700, Analytik Jena AG, Germany).

### Extraction of Cd in different chemical forms

According to Zhang et al. [7], six kinds of Cd with different chemical forms were extracted. The extraction sequence was as follows: (1) 80% ethanol (FE-Cd), extracting inorganic Cd and aminophenol Cd; (2) deionized water (FW-Cd), extracting water-soluble Cd from organic acid complexes and Cd(H_2_PO_4_)_2_; (3) 1 M NaCl (FNaCl-Cd), extracting Cd integrated with pectate and protein; (4) 2% acetic acid (FHAC-Cd), extracting insoluble CdHPO_4_, Cd_3_(PO_4_)_2_ and other Cd-phosphate complexes; (5) 0.6 M HCl (FHCl-Cd), extracting oxalate acid-bound Cd; and (6) Cd in residues (FC-Cd). Approximately 0.5 g of frozen samples were added to the extraction solution at a ratio of 1:10 (w/v), shaken at 25 °C for 22 h, and centrifuged at 5000×*g* for 10 min. The precipitate was resuspended in the same extractive solution twice, shaken at 25 °C for 2 h, and then centrifuged at 5000×g for 10 min. The supernatant was pooled after three centrifugations and evaporated to 1–2 mL on an electric plate. Each form of Cd was digested with HNO_3_:HClO_4_ (4:1, v/v), and the Cd concentration was analyzed by a flame atomic absorption spectrophotometer (ZEEnit 700, Analytik Jena AG, Germany).

### Expression of TaNramp5-a, TaNramp5-b, TaHMA3-a, TaHMA3-b, and TaHMA2

Total RNA was extracted from seedling shoot and root and then used for first-strand cDNA synthesis using the PrimeScript™ RT reagent Kit (TakaRa) in accordance with the manufacturer’s protocol. Gene expression was determined using TB green premix Ex Taq™ II (TakaRa). Relative gene expression was calculated by the 2^−△△Ct^ method. The expression level of Cd_5_Se_0_, as the normalized mRNA level, was set to 1. The primers for *TaHMA2* were obtained from Tan et al. [19], and the primers for *TaNramp5-a*, *TaNramp5-b*, *TaHMA3-a* and *TaHMA3-b* were designed by GenScript Real-time PCR (TaqMan) Primer Design Online (https://www.genscript.com.cn/) based on the mRNA sequences obtained from the Ensembl database (http://plants.ensembl.org/). The primer sequences are shown in Table S1.

### Statistical analysis

$$ \mathrm{Cd}\ \mathrm{accumulation}=\mathrm{Cd}\ \mathrm{concentration}\times \mathrm{dry}\ \mathrm{matter}\ \mathrm{weight} $$

$$ \mathrm{Cd}\ \mathrm{migration}\ \mathrm{coefficient}=\mathrm{Cd}\ \mathrm{concentration}\ \mathrm{in}\ \mathrm{shoot}/\mathrm{Cd}\ \mathrm{concentration}\ \mathrm{in}\ \mathrm{root} $$

The main effects and interactions of Cd and Se were statistically examined by two-way ANOVA using SPSS 7.05 software (Chicago, USA). Tukey’s test was used for multiple comparisons at a 5% significance level (*P* < 0.05).

## Results

### Dry matter weight, cd concentration, and accumulation

Cd treatments had significant effects on dry matter weight in shoot and root (*P* < 0.01; Table S2). Cd and Se treatments had significant effects on Cd concentration and accumulation in shoot and root (*P* < 0.01; Table S3); their interaction had significant effects on Cd concentration (*P* < 0.01; Table S3).

**Fig. 1:**
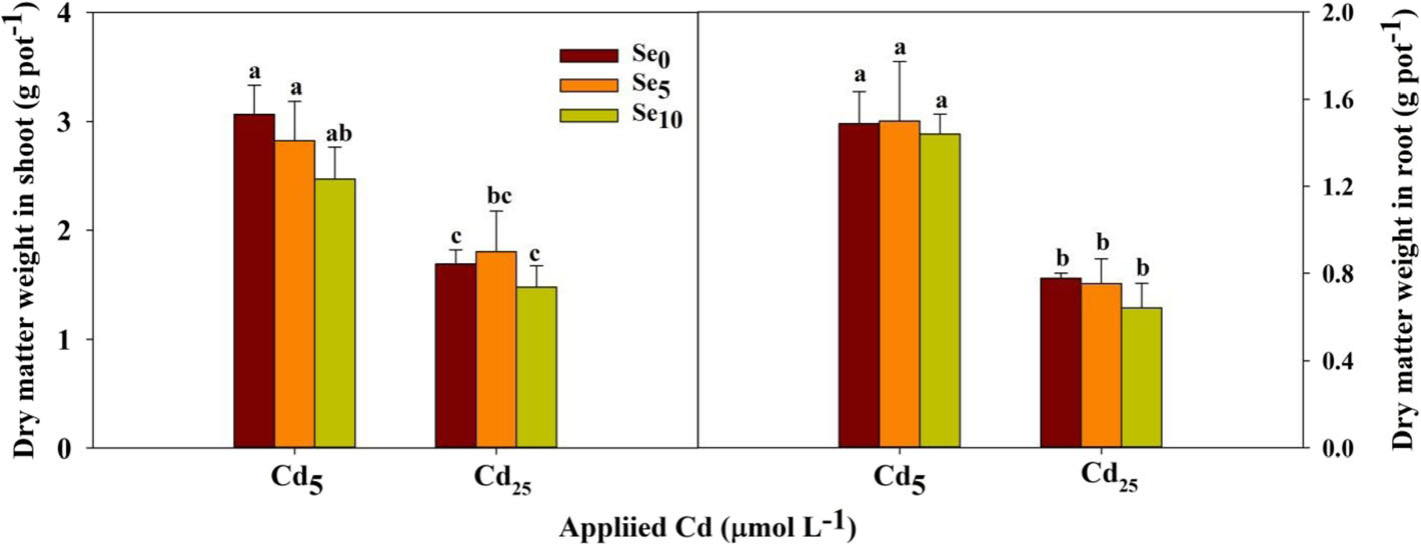
Shoot and root dry matter weights of winter wheat (*Triticum aestivum* cv Zhengmai379) seedlings

Compared with Cd_5_, Cd_25_ significantly decreased the dry matter weight in the shoot and root. Under Cd stress, Se had no significant effect on the dry matter weight of wheat (Fig. 1).

**Fig. 2:**
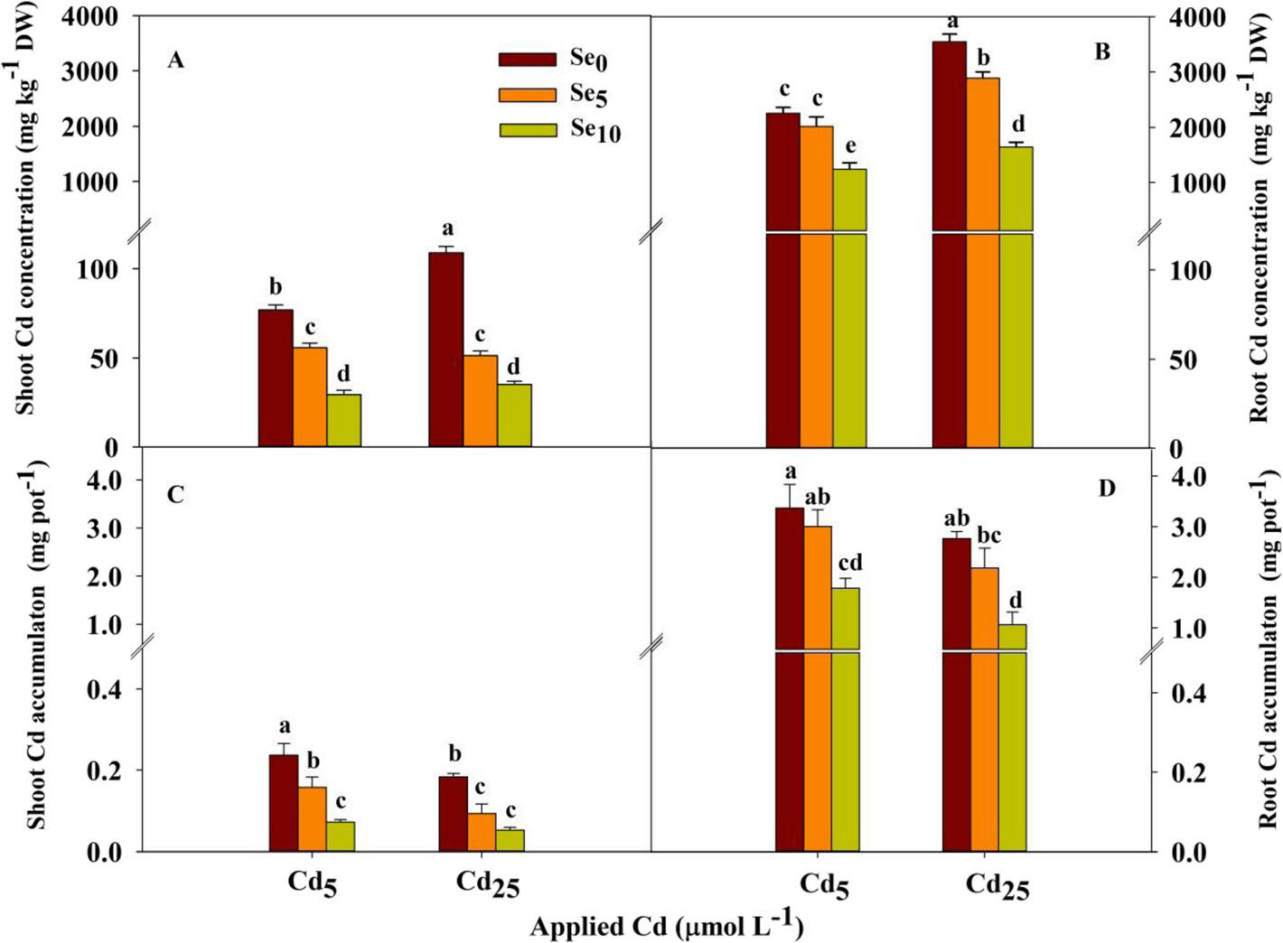
Cd concentration and accumulation in shoot (**a** and **c**, respectively) and root (**b** and **d**, respectively) of winter wheat (*Triticum aestivum* cv Zhengmai379) seedlings

The Cd concentration in root was higher than that in shoot (Fig. 2a and b). In the Se_0_ treatment, the Cd concentration in the shoot and root was significantly increased by increasing the Cd stress level; in the Se_5_ and Se_10_ treatments, the Cd concentration in the root was also significantly increased by increasing the Cd stress level. Compared with Se_0_, Se_5_ and Se_10_ significantly decreased the shoot Cd concentration at each Cd stress level, with the degree of decrease ranging from 27.6 to 67.7% (Fig. 2a). Similarly, Se_5_ and Se_10_ significantly decreased the root Cd concentration at each Cd stress level, with the degree of decrease ranging from 18.6 to 53.6%, except for the lack of an obvious effect of Se_5_ on the root Cd concentration at Cd_5_ (Fig. 2b). The reduction ratio of Cd content in shoot was higher than that in root.

Cd accumulation in root was also higher than that in shoot (Fig. 2c and d). In the Se_0_ and Se_5_ treatments, with the increased Cd stress level, Cd accumulation in shoot was significantly decreased. Compared with Se_0_, Se_5_ and Se_10_ significantly decreased the shoot Cd accumulation at each Cd stress level, with the degree of decrease ranging from 33.3 to 71.6% (Fig. 2c). Se_10_ significantly decreased the root Cd accumulation at each Cd stress level by 46.9 and 61.5% (Fig. 2d).

### Cd migration rate and distribution proportion

Cd and Se treatments had significant effects on the Cd migration rate from root to shoot (*P* < 0.01; Table S3); their interaction had significant effects on the Cd migration rate from root to shoot (*P* < 0.05; Table S3).

**Fig. 3:**
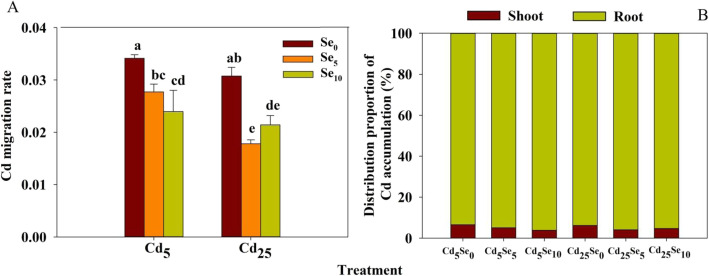
Cd migration rate from root to shoot and Cd distribution proportion of winter wheat seedlings (*Triticum aestivum* cv Zhengmai379)

Compared with the Cd_5_ treatment, the Cd migration rate from root to shoot was significantly decreased by the Cd_25_ treatment at Se_5_ (Fig. 3a). At each Cd stress level, Se_5_ and Se_10_ significantly reduced the Cd migration rate from root to shoot, with the degree of decrease ranging from 18.8 to 30.3%.

At each Cd stress level, Se_5_ and Se_10_ significantly reduced the distribution proportion of Cd accumulation in shoot, but Se_5_ and Se_10_ significantly increased the distribution proportion of Cd accumulation in root (Fig. 3b).

### Root morphology

Cd and Se treatments and their interaction had significant effects on root length, root total surface area and root volume (*P* < 0.01; Table S4). The Se treatments had significant effects on the average root diameter (*P* < 0.01; Table S4).

**Fig. 4:**
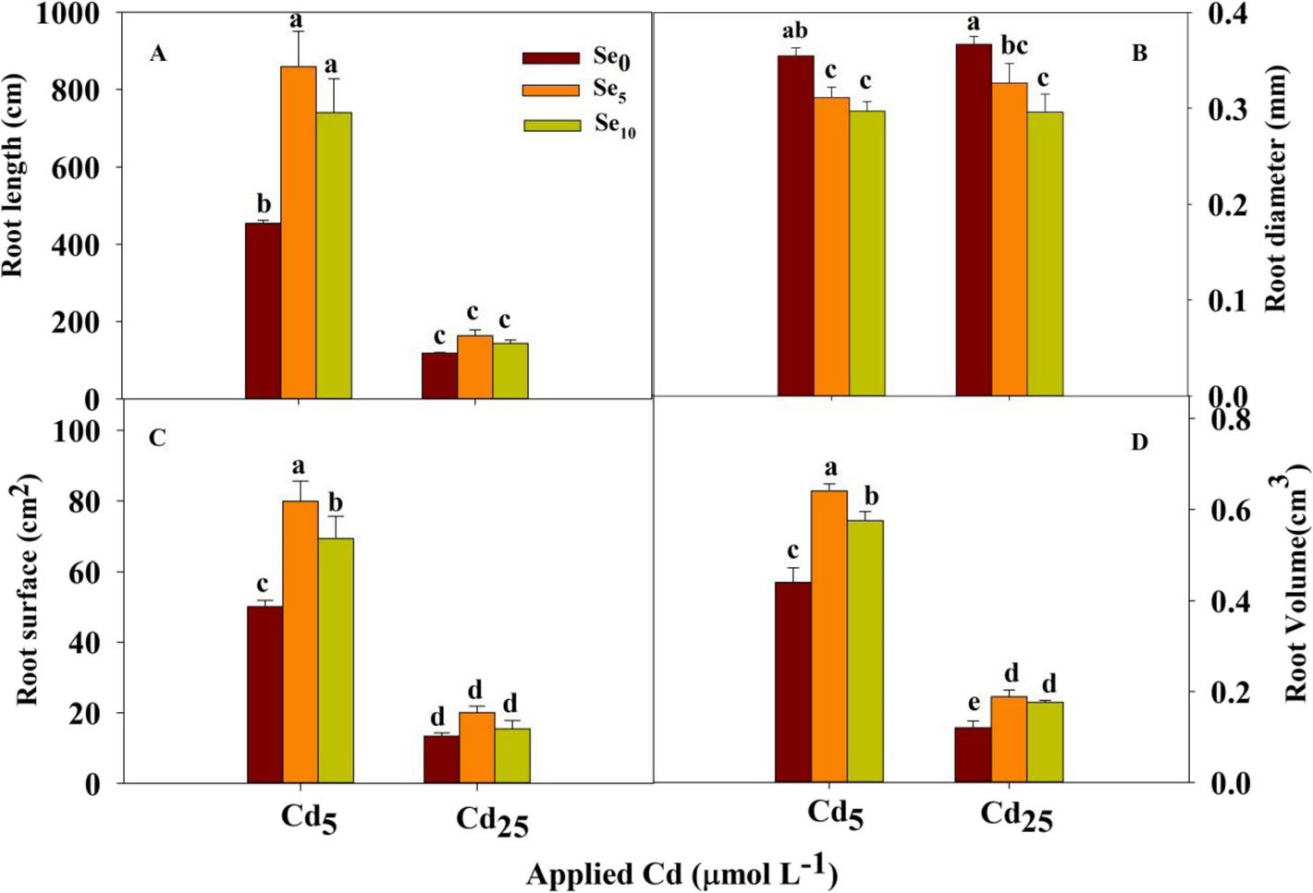
Root morphology parameters of winter wheat seedlings (*Triticum aestivum* cv Zhengmai379)

The root length, root volume and root surface area were reduced significantly with increasing Cd stress (Fig. 4a, c and d). At Cd_5_, Se_5_ and Se_10_ significantly increased the root length, surface area and root volume but decreased the average root diameter in winter wheat, with the degree of decrease or increase ranging from 12.3 to 89.2%. At Cd_25_, Se_5_ and Se_10_ significantly reduced the average root diameter by 11.0 and 19.3%, respectively, but Se_5_ and Se_10_ significantly increased the root volume by 57.2 and 46.9%, respectively.

### Cd subcellular fractionation and distribution

Cd and Se treatments and their interaction had significant effects on the subcellular distribution of Cd in the tissues of wheat seedlings (*P* < 0.01; Table S5).

The Cd concentration in each fraction of shoot and root was significantly increased by increasing the Cd stress level, except for the Cd concentration in the cell wall of shoot at Se_10_, that in the soluble fraction and cell organelles of shoot at Se_5_ and Se_10_ and that in the cell organelles of root at Se_10_ (Table 1). At Cd_5_, the Cd concentration in the cell wall, soluble fraction and cell organelles of shoot were significantly decreased by Se_5_ and Se_10_, with the degree of decrease ranging from 19.0 to 43.2%. Se_10_ significantly decreased the Cd concentration in the soluble fraction and cell organelles of root by 31.3 and 49.3%, respectively. At Cd_25_, Se_5_ and Se_10_ significantly decreased the Cd concentration in the cell wall, soluble fraction and cell organelles of shoot and root, with the degree of decrease ranging from 17.9 to 65.9%.

**Table 1:** Subcellular fractions of Cd in tissues of winter wheat (*Triticum aestivum* cv Zhengmai379) seedlings grown with low (0 μM), medium (5 μM), or high (10 μM) Se supply under low (5 μM) or high (25 μM) Cd stress for 21 d

Treatment		Shoot/(mg·kg^− 1^ DW)			Root/(mg·kg^− 1^ DW)		
Cd (μM·L^− 1^)	Se (μM·L^− 1^)	Cell wall	Soluble fraction	Cell organelle	Cell wall	Soluble fraction	Cell organelle
5	0	3.53 ± 0.07b	11.0 ± 1.05b	2.16 ± 0.32b	21.3 ± 0.71d	138 ± 1.65d	10.7 ± 0.82bc
	5	2.47 ± 0.14c	8.92 ± 0.38 cd	1.60 ± 0.04 cd	19.9 ± 1.83d	128 ± 4.40d	8.57 ± 0.80 cd
	10	2.10 ± 0.12c	7.06 ± 0.33e	1.14 ± 0.23d	16.8 ± 1.45d	95.1 ± 3.95e	5.39 ± 0.53e
25	0	4.61 ± 051a	14.2 ± 0.84a	3.50 ± 0.07a	68.8 ± 0.64a	352 ± 21.9a	22.2 ± 1.55a
	5	3.78 ± 0.04b	10.0 ± 0.46bc	1.93 ± 0.11bc	53.1 ± 3.57b	285 ± 8.39b	13.3 ± 1.58b
	10	2.36 ± 0.18c	7.45 ± 0.25de	1.41 ± 0.16d	45.7 ± 0.66c	188 ± 1.86c	7.58 ± 0.67de

Values are means of three independent replicates (±sd). For each trait, means followed by different letters are significantly different from each other according to two-way ANOVA followed by Turkey multiple comparison (*P* < 0.05)

**Fig. 5:**
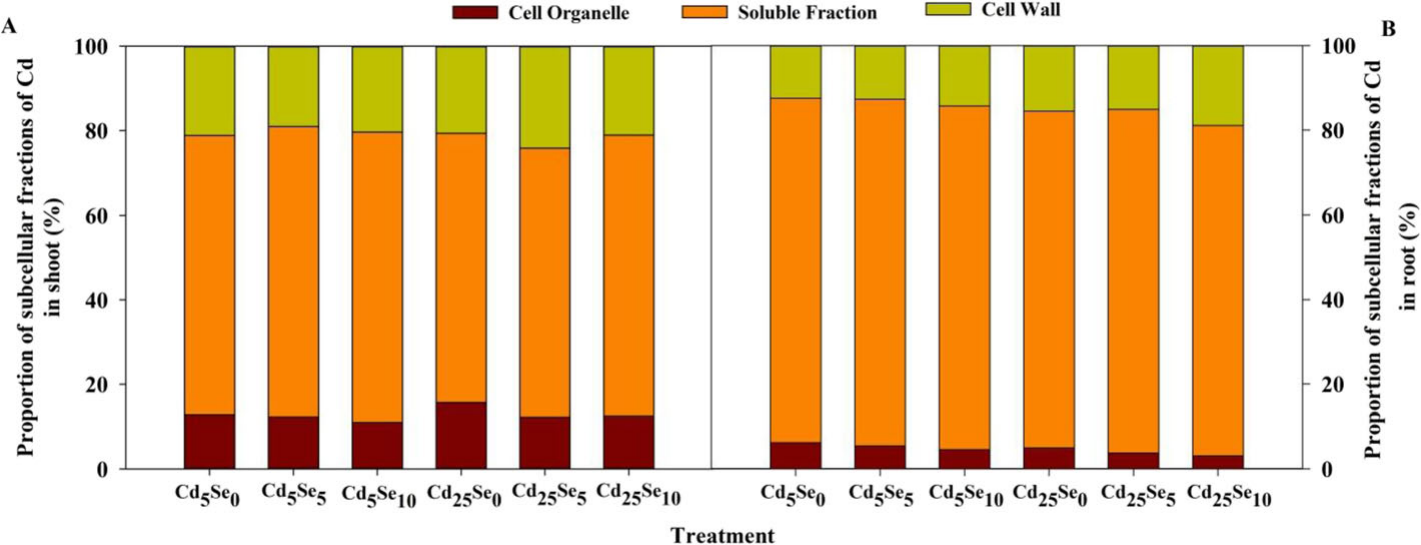
Proportions of Cd in subcellular fractions of winter wheat seedlings (*Triticum aestivum* cv Zhengmai379)

In both the shoot and root, the proportion of Cd in the soluble fraction was higher than that in the cell organelles or cell walls (Fig. 5). The Cd proportion in cell organelles of shoot at Se_0_ and Se_10_, that in the cell wall of shoot at Se_5_ and Se_10_, and that in the cell wall of root at each Se level was increased by increasing Cd stress level; the Cd proportion in the soluble fraction of shoot and root at each Se level, that in the cell wall of shoot at Se_0_, and that in cell organelles of root at each Se level was decreased by increasing the Cd stress level. Se application decreased the Cd proportion in the cell organelles of shoot and root at the two Cd levels, with the degree of decrease ranging from 4.65 to 38.0% (Fig. 5a and b). However, Se application increased the Cd proportion in the soluble fraction of shoot and the Cd proportion in the cell wall of root, with the degree of increase ranging from 1.60 to 21.9%. Se application decreased the Cd proportion in the cell wall of shoot at Cd_5_ but increased its proportion at Cd_25_. Se_5_ increased but Se_10_ decreased the Cd proportion in the soluble fraction of root.

### Cd chemical forms and distribution

Cd treatments had significant effects on FE-Cd, FNaCl-Cd, FHAC-Cd and FC-Cd concentrations in shoot as well as the concentrations of FE-Cd, FW-Cd, FNaCl-Cd, FHAC-Cd, FHCl-Cd and FC-Cd in root (*P* < 0.01 or *P* < 0.05; Table S6). Se treatments had significant effects on FE-Cd, FW-Cd, FNaCl-Cd and FHAC-Cd concentrations in shoot as well as the concentrations of FE-Cd, FW-Cd and FNaCl-Cd in root (*P* < 0.01 or *P* < 0.05; Table S6). There was a significant interactive effect of Se and Cd on FE-Cd concentrations in shoot as well as the concentrations of FE-Cd, FW-Cd, FNaCl-Cd and FHAC-Cd in root (*P* < 0.01; Table S6).

At Se_0_, FE-Cd and FHAC-Cd concentrations in shoot as well as the concentrations of FE-Cd, FW-Cd, FNaCl-Cd, FHAC-Cd and FHCl-Cd in root were significantly increased by increasing the Cd stress level (Table 2). At Se_5_, with an increased Cd stress level, the FE-Cd concentration in shoot as well as the concentrations of FE-Cd, FW-Cd, FNaCl-Cd and FHAC-Cd in root was markedly increased. At Se_10_, with increased Cd stress level, FE-Cd and FNaCl-Cd concentrations in root were significantly increased; but at Se_5_ and Se_10_, the FNaCl-Cd concentration in shoot was dramatically reduced by increasing the Cd stress level. At Cd_5_, Se_5_ and Se_10_ significantly increased the FE-Cd concentration in root but decreased the FNaCl-Cd in shoot and FW-Cd in root, with the degree of decrease or increase ranging from 25.2 to 60.6% (Table 2). At Cd_25_, Se_5_ significantly increased the FE-Cd concentration in shoot but decreased FHAC-Cd in shoot and FW-Cd and FNaCl-Cd in root. Se_10_ significantly decreased FE-Cd, FW-Cd, FNaCl-Cd and FHAC-Cd in shoot and root, with the degree of decrease or increase ranging from 10.1 to 82.0%.

**Table 2:** Chemical forms of Cd in tissues of winter wheat (*Triticum aestivum* cv Zhengmai379) seedlings grown with low (0 μM), medium (5 μM), or high (10 μM) Se supply under low (5 μM) or high (25 μM) Cd stress for 21 d

Tissues	Treatment		Cd/mg·kg-1 DW					
	Cd	Se	FE	FW	FNaCl	FHAC	FHCl	FC
Shoot	5	0	0.62 ± 0.02c	1.27 ± 0.04ab	19.78 ± 1.75a	1.36 ± 0.24b	0.10 ± 0.01a	0.06 ± 0.01a
		5	0.91 ± 0.12bc	1.35 ± 0.10ab	14.79 ± 1.23b	0.74 ± 0.12b	0.11 ± 0.02a	0.06 ± 0.01a
		10	0.80 ± 0.09bc	1.14 ± 0.15ab	12.41 ± 0.94b	0.55 ± 0.08b	0.10 ± 0.02a	0.04 ± 0.01a
	25	0	1.28 ± 0.48b	1.73 ± 0.53a	14.51 ± 0.38b	1.75 ± 0.16a	0.13 ± 0.04a	0.07 ± 0.02a
		5	2.33 ± 0.12a	1.10 ± 0.32ab	12.21 ± 0.44bc	0.83 ± 0.22b	0.10 ± 0.02a	0.07 ± 0.02a
		10	0.51 ± 0.15c	0.97 ± 0.19b	9.59 ± 0.69c	0.71 ± 0.09b	0.13 ± 0.02a	0.07 ± 0.02a
Root	5	0	13.69 ± 0.80e	118.55 ± 3.97c	58.36 ± 4.81de	7.88 ± 1.73b	0.65 ± 0.05b	0.12 ± 0.03ab
		5	17.17 ± 1.28d	80.45 ± 3.96d	67.25 ± 3.35 cd	12.46 ± 0.06b	0.67 ± 0.08b	0.14 ± 0.06ab
		10	21.91 ± 1.86c	46.68 ± 3.13e	56.12 ± 3.52e	13.00 ± 0.91b	0.87 ± 0.13b	0.09 ± 0.04b
	25	0	66.29 ± 0.53a	201.03 ± 5.11a	92.93 ± 1.75a	24.81 ± 4.66a	3.09 ± 1.17a	0.21 ± 0.05a
		5	63.23 ± 1.03a	136.03 ± 3.56b	83.57 ± 1.67b	21.32 ± 4.03a	1.57 ± 0.46ab	0.12 ± 0.04ab
		10	40.81 ± 1.11b	53.14 ± 3.53e	71.91 ± 3.78c	13.36 ± 1.69b	1.90 ± 0.73ab	0.16 ± 0.02ab

Values are means of three independent replicates (±sd). For each trait, means followed by different letters are significantly different from each other according to two-way ANOVA followed by Turkey multiple comparison (*P* < 0.05)

**Fig. 6:**
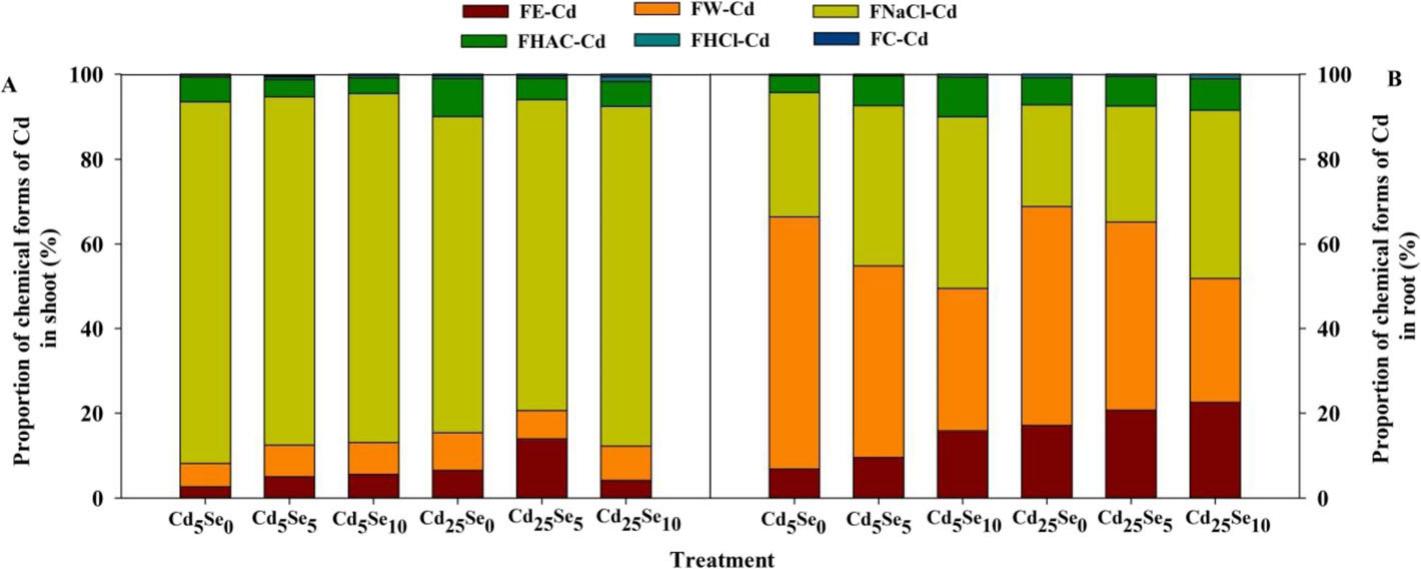
Proportions of Cd in chemical forms of winter wheat seedlings (*Triticum aestivum* cv Zhengmai379)

The Cd proportion in each chemical form in the shoot and root was significantly increased by increasing the Cd stress level, except for FNaCl-Cd in the shoot and root, FW-Cd in root, FHAC-Cd in root at Se_10_, FE-Cd in shoot at Se_10_ and FW-Cd in shoot at Se_5_ (Fig. 6). In root, Se_5_ and Se_10_ increased the proportion of FE-Cd, FNaCl-Cd and FHAC-Cd, with the degree of increase ranging from 9.38 to 135%, but Se_5_ and Se_10_ decreased the proportion of FW-Cd, with the degree of decrease ranging from 14.1 to 43.4%. In shoot, Se increased the proportion of FW-Cd and FE-Cd at Cd_5_ but decreased the FW-Cd and FE-Cd proportions at Cd_25_, FNaCl-Cd and FHAC-Cd proportions at the two Cd levels, except for FE-Cd with Cd_25_Se_5_ treatment and FNaCl-Cd with Cd_25_Se_10_ treatment, with the degree of increase or decrease ranging from 1.48 to 107%.

### Expression of TaNramp5-a, TaNramp5-b, TaHMA3-a, TaHMA3-b and TaHMA2

Cd and Se treatments and their interaction had significant effects on the transcript levels of *TaNramp5-a*, *TaNramp5-b*, *TaHMA3-a*, *TaHMA3-b*, and *TaHMA2* in shoot and root (*P* < 0.05 or *P* < 0.01; Table S7).

**Fig. 7:**
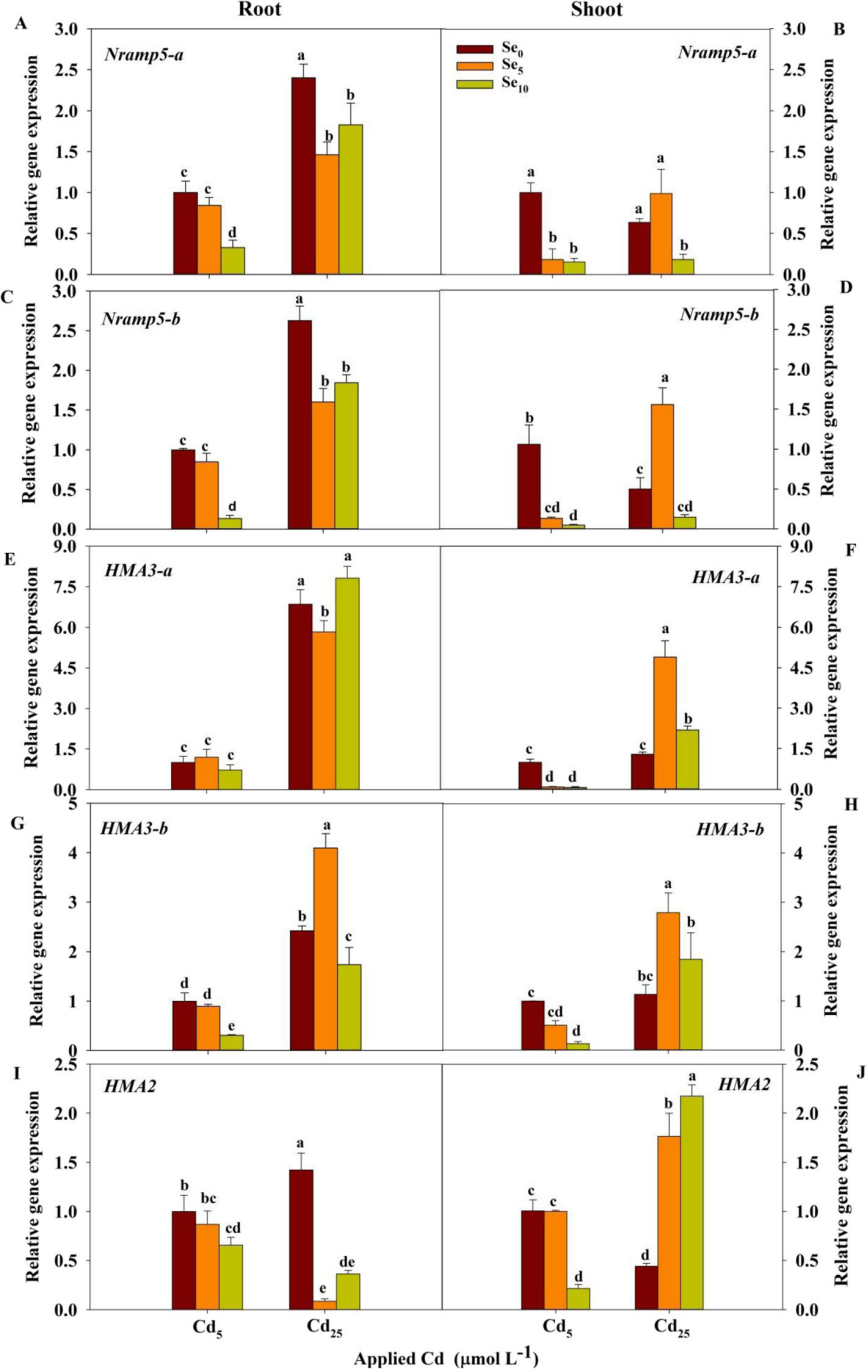
The expression of *TaNramp5-a*, *TaNramp5*-b, *TaHMA3*-a, *TaHMA3*-b and *TaHMA2* in winter wheat seedlings (*Triticum aestivum* cv Zhengmai379)

The transcript levels of *TaNramp5-a*, *TaNramp5-b*, *TaHMA3-a*, *TaHMA3-b* and *TaHMA2* in root were higher than those in shoot, except for the transcript level of *TaHMA2* with Cd_5_Se_5,_ Cd_25_Se_5_ and Cd_25_Se_10_ treatments (Fig. 7). In root, the transcript levels of *TaNramp5-a*, *TaNramp5-b*, *TaHMA3-a* and *TaHMA3-b* were significantly increased by increasing the Cd stress level; increasing Cd stress significantly increased the transcript level of *TaHMA2* at Se_0_ but decreased that at Se_5_ and Se_10_ (Fig. 7a, c, e, g and i). In shoot, increasing Cd stress significantly decreased the transcript levels of *TaNramp5-b* and *TaHMA2* at Se_0_ but increased the transcript levels of the five genes at Se_5_ as well as the transcript levels of *TaHMA3-a*, *TaHMA3-b* and *TaHMA2* at Se_10_ (Fig. 7b, d, f, h and j). At Cd_5_, Se_10_ significantly decreased the transcript levels of *TaNramp5-a*, *TaNramp5-b*, *TaHMA3-b* and *TaHMA2* in root as well as the transcript levels of *TaHMA3-b* and *TaHMA2* in shoot, and Se_5_ and Se_10_ significantly decreased the transcript levels of *TaNramp5-a*, *TaNramp5-b* and *TaHMA3-a* in shoot. At Cd_25_, the transcript levels of *TaNramp5-a*, *TaNramp5*-b and *TaHMA2* in root were significantly decreased by both Se_5_ and Se_10_ treatments; Se_5_ significantly decreased the transcript level of *TaHMA3-a* in root but increased the *TaHMA3-b* transcript level in root and the transcript levels of the five genes in shoot; Se_10_ significantly decreased the *TaHMA3-b* transcript level in root and the *TaNramp5-a* transcript level in shoot but increased the transcript levels of *TaHMA3-a* and *TaHMA2* in shoot.

## Discussion

### Se inhibits Cd absorption by altering root morphology in winter wheat

In our study, the Se supply decreased the Cd concentration and accumulation in both shoot and root (Fig. 2), indicating Se application can inhibit Cd absorption in winter wheat. Huang et al. [20] found that Se application reduced Cd concentration in brown rice by doing a pot experiment, and Lin et al. [21] reported that Se decreases the toxicity and accumulation of Cd in rice by reducing Cd uptake. Plants absorb nutrients mainly through the root system [22]. Many studies have shown that Cd stress can lead to short root length, thick root diameter and a reduced number of lateral root [23]. Our results showed that Se alleviates the toxic effects of Cd on winter wheat root growth, especially under conditions of low Cd stress, showing increased root length, root surface area, and root volume and decreased root diameter with Se application (Fig. 4). However, Ding et al. [24] found that the addition of 0.8 mg L^− 1^ Se to treatments containing 4 mg L^− 1^ Cd increased the length, surface area, volume, and average diameter of root in rice. Root morphology has a great influence on the absorption of minerals [5]. Fine root are the most active part of the root system for mineral absorption [24, 25]. Nazar et al. [26] noted that plant nutrients, such as iron (Fe), manganese (Mn), zinc (Zn) and Cd, compete for the same transporters. Therefore, the inhibited Cd uptake by Se application in this experiment may be related to the decreased root diameter and the increased mineral nutrient uptake by root.

### Se inhibits Cd transport by altering the distribution of Cd in subcellular fractions and by altering Cd chemical forms in winter wheat tissues

Our study suggested Se_5_ and Se_10_ significantly decreased Cd migration rate from root to shoot, the distribution proportion of Cd accumulation in shoot, and the Cd concentration in the cell wall, soluble fraction and cell organelles of shoot and root (Table 1 and Fig. 3). The decreased Cd concentration in subcellular fractions was due to the decreased Cd concentration in winter wheat following Se application. These results also suggested that most of the Cd accumulated in the soluble fraction, followed by the cell wall fraction (Table 1 and Fig. 5). Our results are consistent with the results of Li et al. [27], who found that the majority of Cd was compartmentalized in the soluble fraction (53–75%) and bound to the cell wall (19–42%) in *Agrocybe aegerita*. Cd in the soluble fraction and cell wall is easily chelated and fixed by organic substances, so it is difficult to transfer to other fractions [28]. Li et al. [29] found that Cd in the soluble fraction of wheat root tended to combine with heat-stable protein (HSP), thus reducing the mobility and toxicity of Cd. In addition, vacuoles (involved in the soluble fraction) are considered to accumulate the greatest amount of Cd and are the place where waste and byproducts accumulate [30]. Heavy metals can be separated in vacuoles by binding with various proteins, organic acids and organic bases [31]. In our study, Se application enhanced Cd accumulation in the soluble fraction of shoot (Fig. 5a), indicating that Se use can inhibit Cd migration to other organs, thus alleviating Cd toxicity. The cell wall fraction can bind Cd ions and reduce transport to other parts of the plant, which is the first barrier to protect protoplasts from Cd toxicity [32]. The Cd proportion in the cell wall of root was increased by Se_5_ and Se_10_ (Fig. 5b), suggesting that Se application enhanced Cd accumulation in root to inhibit Cd transport from root to shoot.

Different chemical forms of Cd have distinct migration capacities. For example, compared with undissolved Cd phosphate (FHAC-Cd) and Cd oxalate (FHCl-Cd), inorganic and organic water-soluble Cd (FE-Cd and FW-Cd, respectively) have higher migration abilities and cause greater harm to plant cells [7]. Some studies have shown that FNaCl-Cd plays an important role in the alleviation of Cd toxicity [18, 33]. In our study, Cd was mainly integrated with pectate and protein (FNaCl-Cd) in the shoot and existed in the form of FW-Cd and FNaCl-Cd in the root (Table 2 and Fig. 6). This result indicates that Cd easily migrates from root to shoot in the water-soluble form, but the toxicity of Cd can also be alleviated by converting Cd into undissolved pectate and protein-bound forms. Qiu et al. [34] found that the majority of Cd in both the root and shoot of cabbage was extracted by 1 M NaCl. Some specific polarcompounds contain hydroxyl or carboxyl groups, which can combine with Cd to form a nontoxic complex [18]. Se treatment significantly decreased the total proportion of active Cd (FE-Cd and FW-Cd) but increased the proportion of FNaCl-Cd and FHAC-Cd in root, suggesting that Se reduces the mobility of Cd from root to shoot by promoting the transformation of Cd from the active form to the inactive form in root. The total proportion of active Cd (FE-Cd and FW-Cd) in shoot was decreased by introducing high levels of Se (Se_10_) at Cd_25_, suggesting that a high level of Se can inhibit the mobility of Cd in shoot at a high Cd stress level.

### Downregulation of Cd transporter genes might be responsible for se-decreased Cd accumulation in winter wheat

It is widely believed that Cd enters plant root mainly through the Mn channel protein Nramp5 [35]. *Nramp5* is a member of the Nramp family, located on the plasma membrane of plant root [35]. In our study, the expression of *TaNramp5-a* and *TaNramp5-b* was found in both root and shoot and was significantly increased with increasing Cd concentrations (Fig. 7a, b, c and d), suggesting that Nramp5 might be involved in the absorption and transport of Cd in wheat plants. This finding is in agreement with the results of Ma et al. [36], who showed that the expression of *OsNramp5* was significantly increased at increased Cd concentrations. Tang et al. [37] and Sasaki et al. [35] observed that knockout of *OsNramp5* can significantly reduce the Cd concentration in the root and shoot of rice. In our study, Se use significantly decreased the expression of *TaNramp5-a* and *TaNramp5-b* in shoot at low Cd stress levels (Fig. 7b and d), indicating that Se might inhibit the remobilization of Cd in shoot. In addition, Se treatment significantly decreased the expression of *TaNramp5-a* and *TaNramp5-b* in root (Fig. 7a and c), indicating that the downregulation of *TaNramp5-a* and *TaNramp5-b* by Se may help to decrease Cd uptake in wheat. Cui et al. [38] found that Se pretreatment decreased the expression of *OsNramp5,* thus inhibiting Cd uptake.

Heavy metal ATPases (HMAs) are responsible for the transmembrane transport of cations and play an important role in Cd transport. *HMA3* (heavy metal ATPase3) is located on the vacuole membrane in the root. It is involved in the sequestration of Cd into the vacuoles of root cells, thereby decreasing the transport of Cd to the shoot and reducing the toxicity of Cd [39]. Sasaki et al. [40] reported that overexpression of *OsHMA3* led to decreased root-to-shoot translocation of Cd. In our study, the expression of *TaHMA3-a* and *TaHMA3-b* was found in both root and shoot and was significantly increased with increasing Cd concentrations (Fig. 7e, f, g and h), suggesting that HMA3 might be responsible for the transport of Cd in wheat plants. Se treatment downregulated the expression of *HMA3* in shoot at Cd_5_ but upregulated expression at Cd_25_ (Fig. 7f and h), also indicating that Se can inhibit the remobilization of Cd in shoot by enhancing the sequestration of Cd into vacuoles when Cd stress levels are high. Cui et al. [38] showed that Se pretreatment activated the expression of *OsHMA3,* thus enhancing the transport of Cd into vacuoles.

*HMA2* (heavy metal ATPase2), which is homologous with *HMA3*, belongs to the heavy metal ATPase family. *HMA2* plays a role in the loading of Cd and Zn into xylem and is involved in the root-to-shoot translocation of Cd and Zn [19]. Our results showed that the expression of *TaHMA2* was found in both root and shoot and that it significantly increased with increasing Cd concentrations (Fig. 7i and j). This result suggested that HMA2 might be involved in the transport of Cd in wheat plants. This conclusion is consistent with the results of Tan et al. [19], who showed that the overexpression of HMA2 in wheat and rice increased the root-shoot translocation of Zn/Cd. A recent report showed that lanthanum decreased Cd accumulation in wheat, which may be related to *TaHMA2* downregulation [41]. In our study, Se treatment significantly decreased the expression of *TaHMA2* in root, indicating that the downregulation of *TaHMA2* by Se might contribute to the inhibition of Cd root-to-shoot translocation and the final decrease in Cd accumulation in the shoot of winter wheat. The expression of *TaHMA2* in shoot was significantly increased by Se treatment at Cd_25_ (Fig. 7j), suggesting that Se may promote the remobilization of Cd in shoot by upregulating the expression of *TaHMA2* when the Cd stress level is high. In our studies, chemical forms and transporter processes were affected by the Cd concentration or accumulation. The downregulation of *TaNramp5-a* and *TaNramp5-b* decreased Cd uptake by wheat root and then decreased the Cd content in wheat. The downregulation of *TaHMA2* and the proportion of active Cd in root reduced the migration of Cd to shoot and the distribution of Cd in shoot.

## Conclusions

Our results showed that *TaNramp5*, *TaHMA3* and *TaHMA2* might be responsible for the uptake and transport of Cd in wheat plants. Se application could inhibit Cd absorption and root-to-shoot transport in winter wheat. Our results suggested that Se inhibits Cd absorption by reducing the root diameter and downregulating the expression of *TaNramp5*. Meanwhile, Se inhibits the root-to-shoot translocation of Cd by promoting the distribution of Cd in the cell wall and soluble fractions and in the inactive form in root, as well as downregulating the expression of *TaHMA2* in the root of winter wheat.

## Supplementary Information

**Additional file 1.**

## Data Availability

The datasets generated or analyzed during the current study are available from the corresponding author on reasonable request.

## Abbreviations

FE-Cd: Cd extracted by 80% ethanol
FW-Cd: Cd extracted by deionized water
FNaCl-Cd: Cd extracted by 1 M NaCl
FHAC-Cd: Cd extracted by 2% acetic acid
FHCl-Cd: Cd extracted by 0.6 M HCl
FC-Cd: Cd in residues
