# Supplementary material for: 1Selenium supply alters the subcellular distribution and chemical forms of cadmium and the expression of transporter genes involved in cadmium uptake and translocation in winter wheat (Triticum aestivum)
Source: BMC Plant Biol. 2020 Dec 7;20:550. doi: 10.1186/s12870-020-02763-z (PMC7722431; doi:10.1186/s12870-020-02763-z)
Supplement: Supplementary file 1 — Additional file 1. [file 12870_2020_2763_MOESM1_ESM.docx]

**Supporting Information Tables S1–S7**

**Table S1.** Primers for RT-PCR analysis of the genes.

| **Gene** | **Accession ID** | **Forward primer 5’→3’** | **Reverse primer 5’→3’** |
| --- | --- | --- | --- |
| *TaNramp5-a* | TraesCS4A02G004400.1 | GCGTGGTTACAGGAAAGCAT | AGAAGCCACAGGCAGATCAT |
| *TaNramp5-b* | TraesCS4B02G300600.1 | CTATCTGGATCCCGGCAACT | TCGGATACTCGCTCTTGCAT |
| *TaHMA3-a* | TraesCS5A02G383400.1 | ACATCGCCGTGAGAACAATG | GTCTTGGACTTGCTCTGCTG |
| *TaHMA3-b* | TraesCS5B02G388000.1 | GGCGAGGGCATATATGGAGA | GATCACGTAGCCCATGGAGA |
| *TaHMA2* | JN113581 | TTCCACTGCCTTTCTCCCTC | GGGCATCCGCTTATTTGG |
| *Actin* | TraesCS5D02G132200.1 | GACGCACAACAGGTATCGTGTTG | CAGCGAGGTCAAGACGAAGGATG |

**Table S2** Two-way analysis of variance (ANOVA) of the effects of Cd, Se treatment as well as their interactions on the dry matter weight of winter wheat (*Triticum aestivum* cv Zhengmai379) grown under greenhouse conditions.

| Source of Variation (Treatment) |  | DF |  | Dry matter weight in shoot | | |  | Dry matter weight in root | | |
| --- | --- | --- | --- | --- | --- | --- | --- | --- | --- | --- |
|  |  |  |  | F |  | P |  | F |  | P |
| Cd treatment |  | 1 |  | 71.83 |  | 0.0001 |  | 118.3 |  | 0.0001 |
| Se treatment |  | 2 |  | 3.49 |  | 0.064 |  | 0.742 |  | 0.4968 |
| Cd×Se |  | 2 |  | 0.87 |  | 0.4423 |  | 0.139 |  | 0.8713 |

**Table S3**Two-way analysis of variance (ANOVA) of the effects of Cd, Se treatment as well as their interactions on the Cd concentration and accumulation of winter wheat (*Triticum aestivum* cv Zhengmai379) and Cd migration rate from the roots to shoots grown under greenhouse conditions.

| Source of Variation (Treatment) |  | DF |  | Shoot Cd concentration | | |  | Root Cd concentration | | |  |
| --- | --- | --- | --- | --- | --- | --- | --- | --- | --- | --- | --- |
|  |  |  |  | F |  | P |  | F |  | P |  |
| Cd treatment |  | 1 |  | 74.80 |  | 0.0001 |  | 209.9 |  | 0.0001 |  |
| Se treatment |  | 2 |  | 776.4 |  | 0.0001 |  | 215.0 |  | 0.0001 |  |
| Cd×Se |  | 2 |  | 71.95 |  | 0.0001 |  | 18.91 |  | 0.0002 |  |
| Source of Variation (Treatment) | DF | |  | Shoot Cd accumulation | | |  | Root Cd accumulation | | |  |
|  |  |  |  | F |  | P |  | F |  | P |  |
| Cd treatment  Se treatment  Cd×Se |  | 1 |  | 26.20 |  | 0.0003 | 23.06 | |  | 0.0004 |  |
|  |  | 2 |  | 90.42 |  | 0.0001 |  | 42.95 |  | 0.0001 |  |
|  |  | 2 |  | 2.171 |  | 0.1568 |  | 0.175 |  | 0.8412 |  |
| Source of Variation (Treatment) |  | DF |  | Cd migration rate | | |  |  |  |  |  |
|  |  |  |  | F |  | P |  |  |  |  |  |
| Cd treatment |  | 1 |  | 30.02 | 0.0001 | |  |  |  |  |  |
| Se treatment |  | 2 |  | 45.11 | 0.0001 | |  |  |  |  |  |
| Cd×Se |  | 2 |  | 5.933 |  | 0.0162 |  |  |  |  |  |

**Table S4**Two-way analysis of variance (ANOVA) of the effects of Cd, Se treatment as well as their interactions on the root morphology of winter wheat (*Triticum aestivum* cv Zhengmai379) grown under greenhouse conditions.

| Source of Variation (Treatment) |  | DF |  | Root Length | | |  | \| Average Root Diameter \| \| --- \| | | |
| --- | --- | --- | --- | --- | --- | --- | --- | --- | --- | --- | --- |
|  |  |  |  | F |  | P |  | F |  | P |
| Cd treatment |  | 1 |  | 483.9 |  | 0.0001 |  | 1.879 |  | 0.1955 |
| Se treatment |  | 2 |  | 29.02 |  | 0.0001 |  | 33.87 |  | 0.0001 |
| Cd×Se |  | 2 |  | 19.04 |  | 0.0002 |  | 0.576 |  | 0.5768 |
| Source of Variation (Treatment) |  | DF |  | Root Total Surface Area | | |  | Root Volume | | |
|  |  |  |  | F |  | P |  | F |  | P |
| Cd treatment |  | 1 |  | 787.6 |  | 0.0001 |  | 1933 |  | 0.0001 |
| Se treatment |  | 2 |  | 35.10 |  | 0.0001 |  | 80.96 |  | 0.0001 |
| Cd×Se |  | 2 |  | 14.92 |  | 0.0006 |  | 18.51 |  | 0.0002 |

**Table S5**Two-way analysis of variance (ANOVA) of the effects of Cd, Se treatment as well as their interactions on the subcellular fractions of Cd in tissues of winter wheat (*Triticum aestivum* cv Zhengmai379) grown under greenhouse conditions.

| Source of Variation (Treatment) |  | DF |  | Shoot | | | | | | | | | | | | |
| --- | --- | --- | --- | --- | --- | --- | --- | --- | --- | --- | --- | --- | --- | --- | --- | --- |
|  |  |  |  | Cell wall | | | Soluble fraction | | | | | Cell organelle | | | | |
|  |  |  |  | F |  | P | |  | F |  | P | |  | F |  | P |
| Cd treatment |  | 1 |  | 62.21 |  | 0.0001 | |  | 28.13 |  | 0.0002 | |  | 57.26 |  | 0.0001 |
| Se treatment |  | 2 |  | 90.29 |  | 0.0001 | |  | 111.7 |  | 0.0001 | |  | 115.8 |  | 0.0001 |
| Cd×Se |  | 2 |  | 8.009 |  | 0.0062 | |  | 8.134 |  | 0.0059 | |  | 16.72 |  | 0.0003 |
| Source of Variation (Treatment) |  | DF |  | Root | | | | | | | | | | | | |
|  |  |  |  | Cell wall | | | Soluble fraction | | | | | Cell organelle | | | | |
|  |  |  |  | F |  | P | |  | F |  | P | |  | F |  | P |
| Cd treatment |  | 1 |  | 1846 |  | 0.0001 | |  | 1088 |  | 0.0001 | |  | 148.0 |  | 0.0001 |
| Se treatment |  | 2 |  | 89.59 |  | 0.0001 | |  | 165.9 |  | 0.0001 | |  | 128.6 |  | 0.0001 |
| Cd×Se |  | 2 |  | 43.89 |  | 0.0001 | |  | 54.72 |  | 0.0001 | |  | 30.38 |  | 0.0001 |

**Table S6**Two-way analysis of variance (ANOVA) of the effects of Cd, Se treatment as well as their interactions on Cd chemical forms and distribution in tissues of winter wheat (*Triticum aestivum* cv Zhengmai379) grown under greenhouse conditions.

| Source of Variation (Treatment) |  | DF |  | Shoot | | | | | | | | | | | | |
| --- | --- | --- | --- | --- | --- | --- | --- | --- | --- | --- | --- | --- | --- | --- | --- | --- |
|  |  |  |  | FE | | | FW | | | | | FNaCl | | | | |
|  |  |  |  | F |  | P | |  | F |  | P | |  | F |  | P |
| Cd treatment |  | 1 |  | 33.27 |  | 0.0001 | |  | 0.0120 |  | 0.9143 | |  | 54.41 |  | 0.0001 |
| Se treatment |  | 2 |  | 30.70 |  | 0.0001 | |  | 4.017 |  | 0.0462 | |  | 54.77 |  | 0.0001 |
| Cd×Se |  | 2 |  | 22.90 |  | 0.0001 | |  | 2.925 |  | 0.0923 | |  | 3.200 |  | 0.077 |
| Source of Variation (Treatment) |  | DF |  | Shoot | | | | | | | | | | | | |
|  |  |  |  | FHAC | | | FHCl | | | | | FC | | | | |
|  |  |  |  | F |  | P | |  | F |  | P | |  | F |  | P |
| Cd treatment |  | 1 |  | 7.885 |  | 0.0158 | |  | 2.390 |  | 0.148 | |  | 7.063 |  | 0.0209 |
| Se treatment |  | 2 |  | 55.59 |  | 0.0001 | |  | 0.5870 |  | 0.5714 | |  | 0.6380 |  | 0.5454 |
| Cd×Se |  | 2 |  | 1.454 |  | 0.2721 | |  | 1.436 |  | 0.2761 | |  | 1.204 |  | 0.3338 |
| Source of Variation (Treatment) |  | DF |  | Root | | | | | | | | | | | | |
|  |  |  |  | FE | | | FW | | | | | FNaCl | | | | |
|  |  |  |  | F |  | P | |  | F |  | P | |  | F |  | P |
| Cd treatment |  | 1 |  | 4993 |  | 0.0001 | |  | 677.6 |  | 0.0001 | |  | 199.3 |  | 0.0001 |
| Se treatment |  | 2 |  | 110.3 |  | 0.0001 | |  | 1176 |  | 0.0001 | |  | 23.77 |  | 0.0001 |
| Cd×Se |  | 2 |  | 346.1 |  | 0.0001 | |  | 144.6 |  | 0.0001 | |  | 15.36 |  | 0.0005 |
| Source of Variation (Treatment) |  | DF |  | Root | | | | | | | | | | | | |
|  |  |  |  | FHAC | | | FHCl | | | | | FC | | | | |
|  |  |  |  | F |  | P | |  | F |  | P | |  | F |  | P |
| Cd treatment |  | 1 |  | 45.94 |  | 0.0001 | |  | 27.01 |  | 0.0002 | |  | 5.612 |  | 0.0355 |
| Se treatment |  | 2 |  | 3.234 |  | 0.0753 | |  | 2.434 |  | 0.1297 | |  | 1.381 |  | 0.2885 |
| Cd×Se |  | 2 |  | 13.86 |  | 0.0008 | |  | 3.091 |  | 0.0827 | |  | 2.755 |  | 0.1036 |

**Table S7** Two-way analysis of variance (ANOVA) of the effects of Cd, Se treatment as well as their interactions on the expression of *TaNramp5-a*, *TaNramp5*-b, *TaHMA3*-a , *TaHMA3*-b and *TaHMA2* in tissues of winter wheat (*Triticum aestivum* cv Zhengmai379) grown under greenhouse conditions.

| Source of Variation (Treatment) |  | DF |  | Root | | | | | | | | | | | | |
| --- | --- | --- | --- | --- | --- | --- | --- | --- | --- | --- | --- | --- | --- | --- | --- | --- |
|  |  |  |  | *TaNramp5-a* | | | *TaNramp5-b* | | | | | *TaHMA3-a* | | | | |
|  |  |  |  | F |  | P | |  | F |  | P | |  | F |  | P |
| Cd treatment |  | 1 |  | 232.8 |  | 0.0001 | |  | 578.8 |  | 0.0001 | |  | 1152 |  | 0.0001 |
| Se treatment |  | 2 |  | 26.06 |  | 0.0001 | |  | 74.85 |  | 0.0001 | |  | 6.336 |  | 0.0132 |
| Cd×Se |  | 2 |  | 13.10 |  | 0.001 | |  | 29.22 |  | 0.0001 | |  | 16.83 |  | 0.0003 |
| Source of Variation (Treatment) |  | DF |  | Root | | | | | | | |  |  |  |  |  |
|  |  |  |  | *TaHMA3*-b | | | *TaHMA2* | | | | |  |  |  |  |  |
|  |  |  |  | F |  | P | |  | F |  | P | |  |  |  |  |
| Cd treatment |  | 1 |  | 451.7 |  | 0.0001 | |  | 15.30 |  | 0.0021 | |  |  |  |  |
| Se treatment |  | 2 |  | 80.30 |  | 0.0001 | |  | 74.76 |  | 0.0001 | |  |  |  |  |
| Cd×Se |  | 2 |  | 38.75 |  | 0.0001 | |  | 39.75 |  | 0.0001 | |  |  |  |  |
| Source of Variation (Treatment) |  | DF |  | Shoot | | | | | | | | | | | | |
|  |  |  |  | *TaNramp5*-a | | | *TaNramp5*-b | | | | | *TaHMA3*-a | | | | |
|  |  |  |  | F |  | P | |  | F |  | P | |  | F |  | P |
| Cd treatment |  | 1 |  | 5.293 |  | 0.0401 | |  | 22.48 |  | 0.0005 | |  | 376.1 |  | 0.0001 |
| Se treatment |  | 2 |  | 31.00 |  | 0.0001 | |  | 50.15 |  | 0.0001 | |  | 52.86 |  | 0.0001 |
| Cd×Se |  | 2 |  | 25.37 |  | 0.0001 | |  | 74.69 |  | 0.0001 | |  | 111.1 |  | 0.0001 |
| Source of Variation (Treatment) |  | DF |  | Shoot | | | | | | | |  |  |  |  |  |
|  |  |  |  | *TaHMA3*-b | | | *TaHMA2* | | | | |  |  |  |  |  |
|  |  |  |  | F |  | P | |  | F |  | P | |  |  |  |  |
| Cd treatment |  | 1 |  | 103.8 |  | 0.0001 | |  | 172.4 |  | 0.0001 | |  |  |  |  |
| Se treatment |  | 2 |  | 9.459 |  | 0.0034 | |  | 51.55 |  | 0.0001 | |  |  |  |  |
| Cd×Se |  | 2 |  | 22.47 |  | 0.0001 | |  | 177.1 |  | 0.0001 | |  |  |  |  |
